# Lessons Learned From Building a Data Platform for Longitudinal, Analytical Use Cases and Scaling to 77 German Hospitals: Implementation Report

**DOI:** 10.2196/69853

**Published:** 2025-09-12

**Authors:** Markus Bockhacker, Peter Martens, Clara von Münchow, Sarah Löser, Rosita Günther, Ralf Kuhlen, Olaf Kannt, Sebastian Ortleb

**Affiliations:** 1 Helios Kliniken, Friedrichstraße 136, Berlin, 10114, Germany, 49 305213210; 2 Helios IT Service GmbH, Berlin, Germany; 3 Helios Health Institute, Berlin, Germany

**Keywords:** research platform, secondary data use, data science, Hadoop, broad consent, medical informatics

## Abstract

**Background:**

Increasing adoption of electronic health records (EHRs) enables research on real-world data. In Germany, this has been limited to university hospitals, and data from acute care hospitals below the university level are lacking. To address this, we used established design patterns to build a data platform that aggregates and standardizes pseudonymized EHR data with patients’ consent.

**Objective:**

We report on the design and implementation of the research platform, as well as patient participation and lessons learned during the scaling of the platform, to incorporate real-world data (with participant consent) from 77 hospitals into a unified data lake.

**Methods:**

Due to variations in EHR adoption, IT infrastructure, software vendors, interface availability, and regulatory requirements, we used an agile development cycle that involves constant, incremental standardization of data. We implemented a layered lambda infrastructure built on Apache Hadoop. Decentralized connectors ensured data minimization and pseudonymization.

**Implementation (Results):**

We successfully scaled our data model both vertically and horizontally in 77 hospitals. However, we encountered issues during the scaling of real-time data pipelines and IHE (Integrating the Healthcare Enterprise) interfaces. During the first 2 years, patients were asked to consent to secondary data use 1,475,244 times during inpatient admission. We registered 1,023,633 broad instances of consent (consent rate 70.2%).

**Conclusions:**

Patients are generally willing to provide consent for secondary use of their data, but obtaining consent requires considerable effort. Building a research data platform is not an end goal, but rather a necessary step in collecting and standardizing longitudinal data to enable research on real-world data. Through the combination of agile development, phased rollouts, and very high levels of automation, we have been able to achieve fast turnaround times for incorporating user feedback and are constantly improving data quality and standardization.

## Introduction

Adoption of electronic health record (EHR) systems has continuously increased in Germany over the past 20 years [1], mainly driven by billing requirements and mandatory digital reporting for quality management. EHR data hold promise for medical quality assurance as well as clinical and health services research [2-4], referred to as secondary data use. However, data silos limit accessibility and hinder cross-institutional analyses.

In 2018, the German Ministry of Health initiated a national health data platform for research on real-world data collected from university hospitals. As part of the German Medical Informatics Initiative, several consortia received funding to develop Health Level Seven (HL7) Fast Healthcare Interoperability Resources (FHIR) models [5], governance processes [6-8], and a broad consent standard for secondary data use [9]. Although European Union and national laws regulate secondary use, consent is typically required to use such data for medical research in Germany [10]. The Helios Hospital group, operating more than 89 acute care hospitals and 230 outpatient care centers in Germany as of December 2024, has championed voluntary data collection and reporting for quality management, enabling cross-institutional research and peer review [11-14]. Secondary analysis of claims data across our hospital network has already generated insights into admission rates and mortality during the COVID-19 pandemic [15-17]. Existing data centers, network infrastructure, and a unified IT department enable us to build a data platform for secondary data analysis of EHR, quality assurance, and claims datasets in-house.

## Methods

### Aims and Objective

We describe the design and implementation of a cross-institutional, multidomain data and analytics platform with a modular data model across our organization. While similar platforms have been described for one or a few institutions [18-24], integrating multiple hospitals with differing EHR systems presents unique, yet undocumented challenges [25]. We emphasize highlighting these challenges and presenting our lessons learned for scaling a data platform across diverse software systems, regions, and data domains. Specific research projects are beyond this paper’s scope. We used iCHECK-DH (Guidelines and Checklist for the Reporting on Digital Health Implementations) [26] to structure this report.

### Requirements Engineering

Upon project initiation, we assembled a cross-functional team comprising research, compliance, project management, and engineering members to ascertain requirements. We considered both data and user perspectives in the design. We conducted an initial high-level data discovery process and interviews with medical, technical, and regulatory subject matter experts. Though unstructured, the interviews helped define key requirements and agile personas (Table 1) to guide development.

**Table 1.** Agile personas to guide developing the data platform.

	Persona “Heather”	Persona “Mere”	Persona “Dave”
Function	Health services researcher	Medical researcher	Data engineer/scientist
Background	Epidemiologist with strong statistical background	Physician-scientist with advanced statistical knowledge	Computer scientist
Reasoning	Ensure and improve quality of treatment of patients nationally	Perform and publish peer-reviewed medical research	Advise business users and monitor data quality
Objectives	Facilitate change in processes or standard operating procedures and guidelines	Perform retrospective cohort studies or decide on feasibility of prospective clinical trials	Provide data to users
Example problem	Are antibiotics dosed correctly and routinely monitored during therapy?	Do courses of therapy differ between urban and rural regions?	Does the amount of data correlate to the number of patients who consented to secondary data use?

### Infrastructure and Software

Because of regulatory requirements, we implemented an on-premise data platform instead of a cloud-based “platform-as-a-service” solution. Our requirements mandated integration of multiple data types, from tabular data to binary blobs. We selected a lambda architecture based on the Apache Hadoop stack, as discussed by McPadden et al [18], enhanced by Python and R notebooks for rapid analytics [27]. To reduce operational complexity, we used a Hadoop distribution offering cluster configuration, monitoring tools, and vendor support. Our setup operates in fully kerberized mode, requiring authentication for each data operation and generating auditable logs.

We provide three tools to interact with the data lake:

1. Apache Hue with access to Impala-SQL, Hive-SQL, H-Base, and the Hadoop Distributed File System
2. A hosted data science environment with containerized runtimes for Python, R, and custom data applications
3. Hosted BI-Tools for self-service analytics using dashboards

### Consent Management

Participation is opt in and requires written consent. This consent is modular, covering broad consent for research on real-world data and contact for clinical trial recruitment. While similar to the Medical Informatics Initiative broad consent, our version excludes modules like biobanking or genetic data, as these domains are rarely available in our hospitals.

We developed a consent management system from scratch to handle modular consent with validity periods. Two interfaces (HL7v2 and REST-API via HTTPS) connect the back end to EHR-integrated front ends, minimizing clicks and avoiding disruptions. Consent information is stored in a multitenant database linked to case number and a validity period. Authorized staff can trigger withdrawals; stored procedures remove expired or withdrawn consent daily to maintain an up-to-date list.

### Extract, Transform, Load

**Figure 1.**
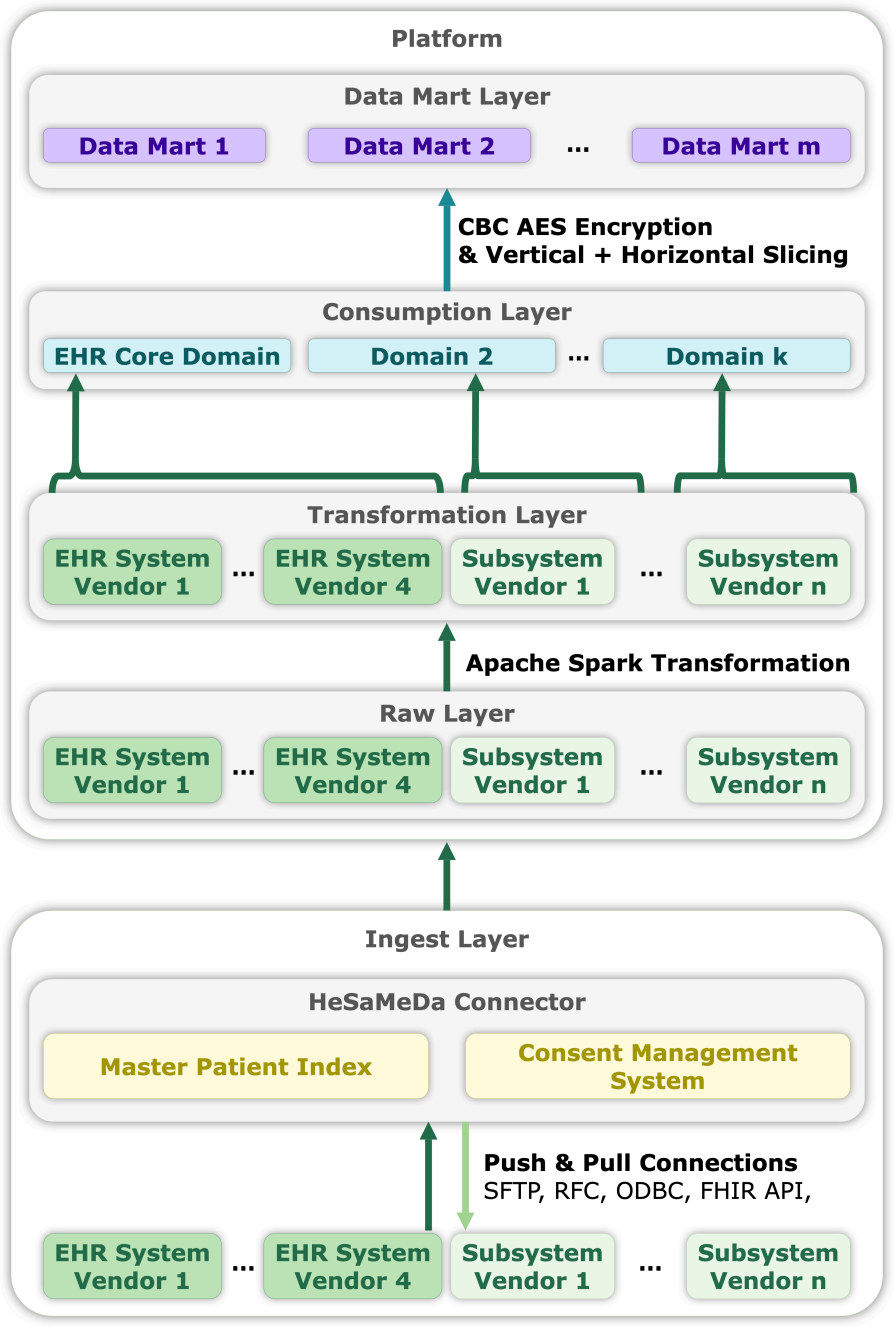
Data flows from EHR systems through the CONNECTOR into a data lake. Spark pipelines transform data through a layered structure. Users access data in horizontally and vertically sliced data marts. AES: advanced encryption standard; API: application programming interface; CBC: cipher block chaining; EHR: electronic health record; FHIR: Fast Healthcare Interoperability Resources; HeSaMeDa: Helios Safe Medical Data (Platform); ODBC: open database connectivity; RFC: remote function call; SFTP: secure file transfer protocol.

Extract, transform, load processes for EHR systems have been described previously [28-30]. This paper details our approach to integrating individual hospitals into a unified data lake. We also outline our data modeling and standardization processes. Figure 1 summarizes the data flow.

EHR adoption levels vary across German hospitals, including within our group, as measured by the electronic medical record adoption model level [31]. For historical reasons, Helios uses EHR systems from different vendors with different subsystems. Hence, we developed an ingest layer supporting diverse formats and delivery methods.

The connector is a containerized Linux system configurable for single- or multitenant use. Java and Python runtimes filter, minimize, and pseudonymize the data before pushing it to the Hadoop cluster.

### Data Minimization and Pseudonymization

Regulatory requirements mandate that data be minimized to research-relevant information and pseudonymized once they leave the regulatory hospital domain [32].

Preferably, source systems would filter for patient consent, pseudonymize records, and push these to the platform. Some EHR systems allow custom business logic via remote function calls to meet these requirements. FHIR application programming interfaces (APIs) often support minimization through custom profiles that exclude personally identifiable attributes (eg, MedicationAdministration.performer). Systems without API access require vendor support for custom interfaces or queries. Additional minimization and pseudonymization are typically applied within the connector for compliance.

Helios treats patients across inpatient and outpatient settings and across multiple hospitals. To ensure correct longitudinal mapping, we implemented a unique pseudonymous identifier using a dedicated master patient index (MPI). The MPI, based on validated probabilistic criteria [33], consolidates records via the IHE (Integrating the Healthcare Enterprise) protocol (Patient Identifier Cross-referencing [34]) into a unique pseudonymous patient entry. This identifier is transmitted back to the connector to replace the original patient ID. Before granting data access to researchers, we add a second layer of pseudonymization to further reduce reidentification risk across projects [32].

### Data Modeling and Transformation

We designed a simple core data model and a four-layer transformation architecture:

1. A raw layer keeps ingested data in append-only store, without relational schema constraints.
2. A transformation layer uses Apache Spark to process raw data in pipelines grouped by source system. Code is continuously deployed via a continuous integration and continuous deployment (CI/CD) pipeline and transforms data into our data model. Transformations run in batches by data source similarity, not by hospital, reducing pipeline complexity.
3. The consumption layer unifies data from different EHR systems into a single EHR core domain, enriches it with semantic information like readable text for codes, and performs data quality and plausibility checks.
4. The data mart layer offers researchers sliced subsets of the consumption layer. Data marts can be stored as checksummed archives to ensure reproducible analyses.

**Figure 2.**
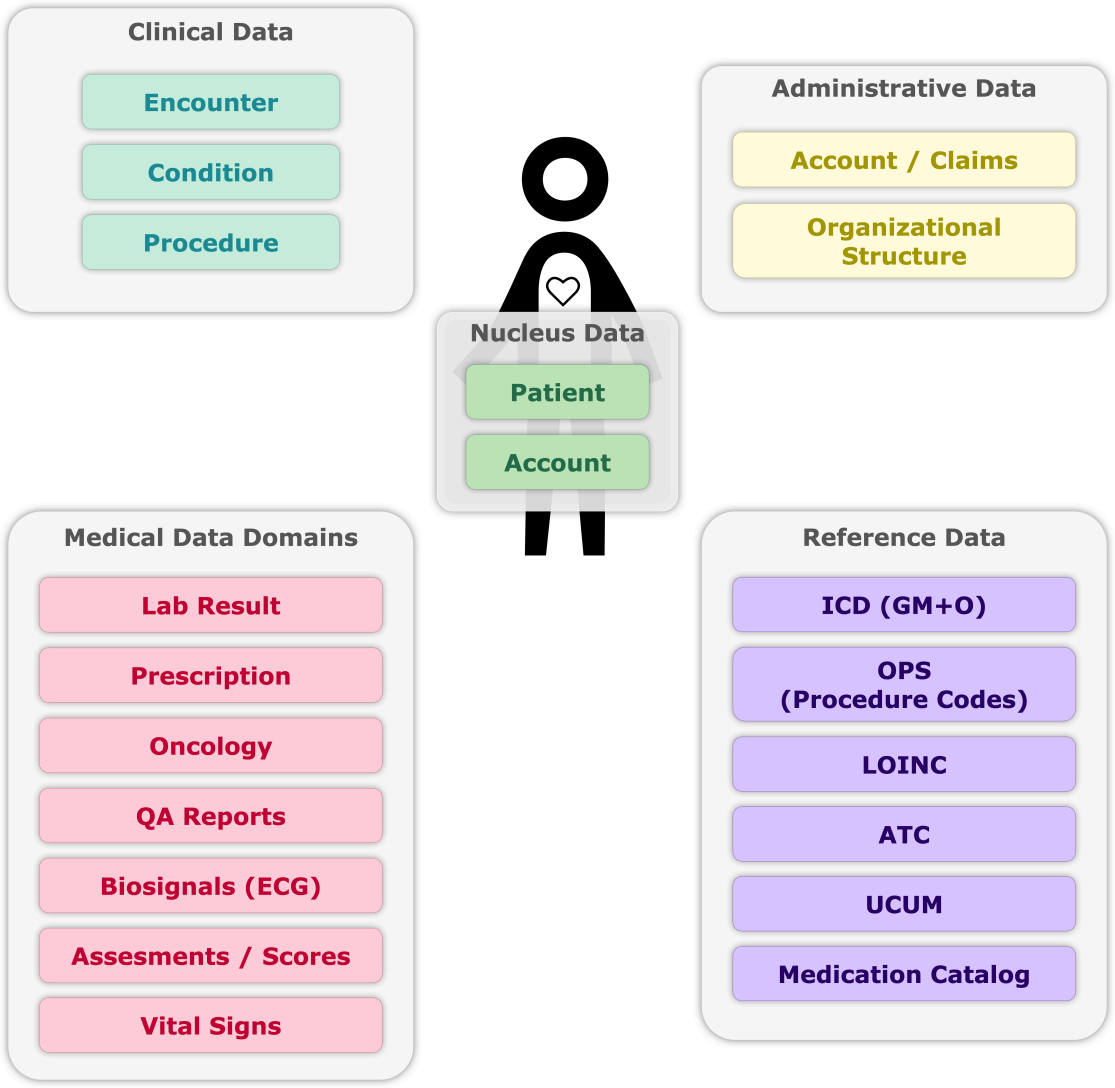
Data domains. Reference data are used to enrich and standardize data in other data domains and are used mainly for transformation processes. Other data domains contain pseudonymized data from patients who gave consent for secondary data use. ATC: Anatomic Therapeutic Chemical (classification); ECG: electrocardiogram; *ICD*: *International Classification of Diseases*; *ICD GM*: *ICD German Modification*; *ICD O*: *ICD Oncology*; LOINC: Logical Observation Identifiers Names and Codes; OPS: operation and procedure codes (in Germany); QA: quality assurance; UCUM: Unified Code for Units and Measurements.

The data model centers on structured data compatible with the HL7 FHIR resources for patients and accounts (Figure 2), enabling representation of inpatient and outpatient visits linked to constant patient objects. Our model closely follows the core dataset of the German Medical Informatics Initiative [35] with deviations for data privacy reasons (eg, reducing birth date to birth year) and data availability (eg, excluding employee data like *requester and performer* in *MedicationRequest* or missing *device* attribute of the *observation*). Two design principles were used: preserving raw data and prioritizing speed over complete standardization. Because most users are experienced in statistics and data analysis, we iteratively refined standardization based on insights from analytical projects. Examples include calculating patient age at admission and converting lab results to international standards like the Unified Code for Units of Measure. Raw values are provided alongside transformed data to maintain trust, though an interactive data lineage is not available.

### Development Workflow and Process Scheduling

**Figure 3.**
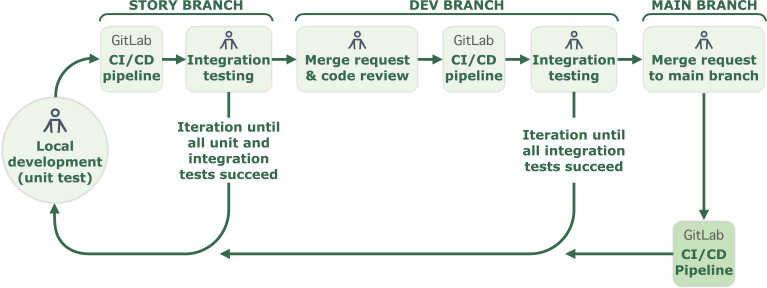
Agile development workflow using continuous integration and continuous deployment (CI/CD). PySpark code from story branches is tested and evaluated multiple times before it can be merged into production. With each iteration the level of scrutiny increases, from unit tests to integration into tests and manual code review.

All transformation code is stored in a versioned repository (git), with each feature developed in its own branch, per agile practices. A CI/CD pipeline runs unit and integration tests on each commit using reproducible Docker builds, with a focus on conditional branching. Code is deployed to production only via the CI/CD pipeline (Figure 3) after tests and manual review. Execution and scheduling, including dependencies, are managed via directed acyclic graphs in Apache Airflow. After a successful run, the story owner conducts a user acceptance test before sign-off and archiving of the feature branch.

### Governance

Data governance follows German guidelines for secondary data analysis [36]. The multidisciplinary Usage and Access Committee (UAC) grants data access per use case, enforced at column and row level by user- or group-based policies in Apache Atlas. Reidentification of patients requires approval from researchers, the ethics committee, and the UAC, typically for clinical trial inclusion, and is performed only at the data-providing hospital, to preserve data sovereignty.

## Implementation (Results)

### Consent Management

**Figure 4.**
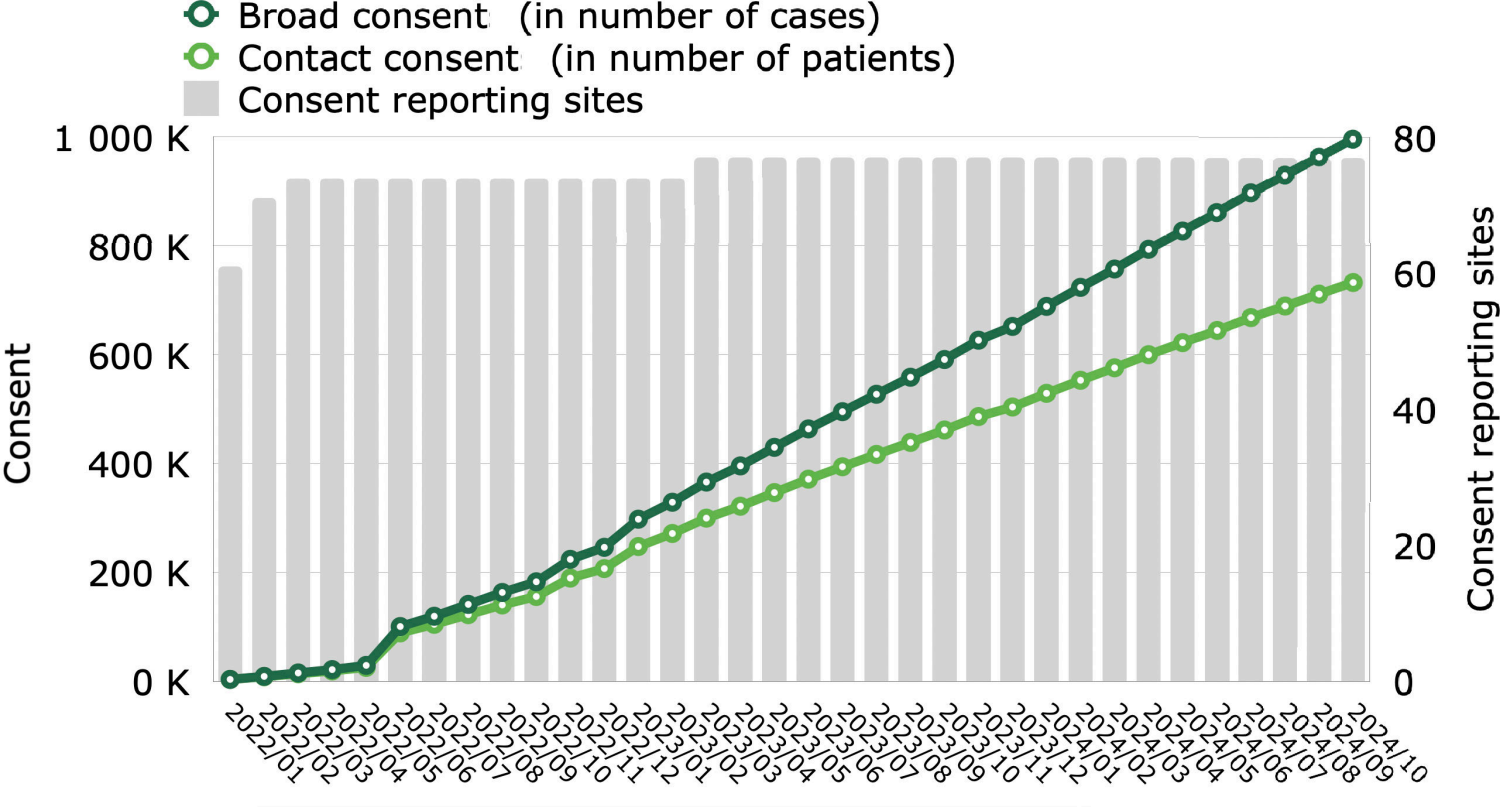
Broad consent and contact consent since project launch. Consent infrastructure, as measured by the number of hospitals (sites) reporting consent, was rolled out faster than data infrastructure (see also Figure 5).

**Figure 5.**
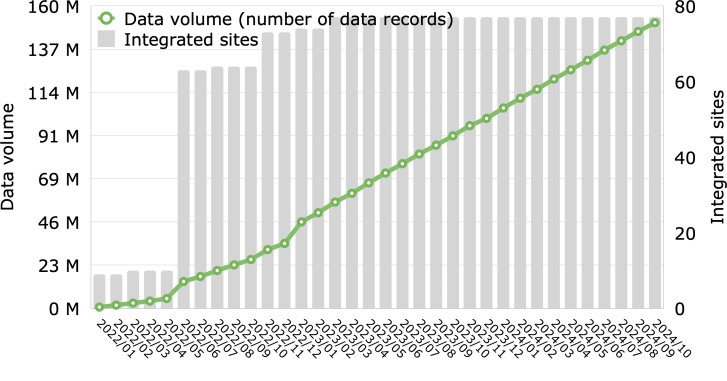
Data volume over time, as measured by record counts in the consumption layer in relation to the integrated sites (hospitals), which illustrates a staged rollout and scaling of data infrastructure.

From January 2022 to July 2023, the consent management system was implemented in 77 hospitals (Figure 4 and Figure 5). Ten hospitals were excluded due to their specialization (psychiatric) or incomplete network integration.

In general, EHR updates occur in cycles within our hospital network. Consent management, which is integrated into the administrative user interface, requires thorough testing before deployment, as updates have to wait until the next cycle. Scaling was rapid: 61 facilities were integrated within the first month and 10 more within the second, and there were a total of 74 within 3 months. Staff received training during the rollout, yet in the initial 6 months, only 40% of patients were asked for consent, with a variation of plus or minus 24% across hospitals. Additional training boosted the rate to 63% by the end of 2023.

Rapid consent integration caused a significant load on the MPI during peak patient admission in the morning. To avoid time-outs, the Patient Identifier Cross-referencing interface was wrapped in an HTTPS API behind a high-availability load balancer.

Over a 24-month period, 1,475,244 patients were asked for consent during admission. In total, we received 1,023,633 instances of broad consent (consent rate: 70.2%), and 732,485 (71.6%) of these also included agreement to be contacted by researchers.

Notably, consent was not uniformly distributed: 714521 (69.8%) of instances stemmed from planned admissions, 217583 (21.3%) from unplanned admissions through the emergency department (ED), and 91529 (8.9%) from outpatient cases in the ED.

### Data Volume

After 24 months, the consumption layer held approximately 15.1 billion records (averaging 102 unique medical data points per case), as shown in Figure 5. This amounted to approximately 14.1 terabytes of data as of October 2024. The ingest layer managed on average 1.9 million HL7 messages and 45,378 FHIR bundles per year, per hospital.

### Data Modeling and Transformation

An iterative development approach results in frequent, additive changes to the data model, which are difficult to communicate to users. Therefore, we developed an interactive data catalog as a web application that displays the current state of all tables in the Hive Metastore.

We implemented an automated data quality framework to ensure data model conformity, assess data relationships, and detect outliers. This helped identify point-of-care devices reporting lab results in nonstandard units during LOINC (Logical Observation Identifiers Names and Codes) mapping [37].

Since transformations evolve, we implemented data version control [38] to store versioned code and data for reproducibility. It worked very well, but required familiarity with git and command line interfaces.

### Lessons Learned

#### Infrastructure Scaling

The Hadoop ecosystem handles structured, semistructured, and unstructured data. Due to data minimization, data volumes grow more slowly than the compute demand, driven mainly by standardization and data quality checks. Therefore, we added another physical host to the cluster in the first year.

The consent rollout required addressing regulatory compliance, seamless technical integration into patient management systems, and an organizational transition to embed consent into the admission process, which presented unexpected challenges. A stable consent rate of 70% showed strong patient willingness to support research.

#### Strategies for Scaling the Data Lake

In summary, a 2-phase data integration strategy, starting with a few hospitals, has proven effective. The initial phase facilitates refinement of interfaces, data modeling, and transformation processes, laying the foundation for seamless integration with a larger number of hospitals during subsequent scaling. Scaling the consent management system prior to data integration also proved beneficial, ensuring a consistent backlog of data for integration into the data lake.

Notably, as our data ingestion interfaces matured, technical integration of new data domains like ICU or cancer registries accelerated. However, full data model integration still requires substantial input from subject matter experts.

#### Batched Data Scales More Easily Than Streaming Data

Scaling adversely affected our MPI and HL7v2/IHE data ingestion, as these systems rely on an existing communication infrastructure that is not readily scalable. Future real-time data integration will likely shift toward local clinical data repositories for preprocessing, rather than introducing new streaming pipelines with technologies like Apache Kafka.

#### Interoperability and Standardization

Standardization, even for basic resources, presents difficulties, as transformations impact semantics and may lead to permanent information loss, despite agreement on rules by involved parties (ie, subject matter experts and data engineers).

Divergence in target values across different EHR installations poses risks to information integrity, notably during initial HL7 message processing due to profile limitations excluding certain HL7 “Z-segment” permutations. Consequently, a “raw-data first” policy was implemented to mitigate future data loss, albeit at the expense of increased bandwidth and storage capacity needs. Standardization can also result in decreased precision, particularly when transforming observations that were recorded without adhering to standards like LOINC [37].

#### Automation

Automation is essential to enhance efficiency, accuracy, and productivity. Versioned code and reproducible builds from the CI/CD pipeline helped to quickly revert changes that turned out to be suboptimal prior to production deployment. We also had substantially more success in onboarding new personnel onto automated, rather than manual, processes. We argue this learning is especially valuable for research institutions, which experience frequent personnel changes among graduate students and postdoctoral researchers.

### Unintended Consequences

With 70% of cases in our dataset stemming from planned admissions, ED cases were underrepresented, introducing a selection bias. This disparity compromises the validity of results extrapolated to the ED setting and may distort the performance metrics of predictive models and risk stratification algorithms, possibly resulting in suboptimal outcomes. Thus, meticulous methodological scrutiny, starting in the design phase of each project, is required to mitigate these biases when using this dataset for translational research.

To enhance unbiased subsampling and improve generalizability, we categorized admission criteria for accounts and encounters. Nevertheless, our aim is to seek consent from every patient, irrespective of admission type, ensuring dataset balance. Obviously, in urgent medical situations, obtaining consent may not be feasible for patients who are distressed or have an altered mental state. A potential solution is to separate consent from administrative admission, seeking it during the inpatient stay instead, although this approach would demand extra resources. Unfortunately, vulnerable patient groups (eg, homeless or uninsured patients) will also be underrepresented in our dataset, since most of their interaction with the medical system is through the ED. Ethical implications extend beyond consent collection. The potential for algorithmic bias in medical research based on our dataset must be carefully considered. Researchers using our platform are required to report on the demographic composition of their study populations and explicitly address potential biases in their analyses.

## Discussion

We hope to inspire other private hospital networks to build data lakes. Adhering to best practices as defined by national and international research consortia is key to ensure compatibility and to allow for integration with other research consortia in the future. Combining data from academic and nonacademic institutions and health care providers could enable longitudinal analysis of patients across multiple health care sectors, and the German Medical Informatics Initiative has begun branching out into nonacademic health care providers via progress hubs [39].

Our approach centralizes data from all participating hospitals after integration, whereas the German Medical Informatics Initiative operates local data integration centers at every participating hospital or within smaller hospital clusters. Decentralized data repositories provide local autonomy and resilience against system failures but demand infrastructure and personnel investment at every site. A decentralized approach seems ideal for academic institutions because local data expertise can be used for local research projects and education of computer science students as well as physician scientists receiving specialization training. For hospital networks below the level of university medical centers, centralization might be preferable, especially if there are already shared IT resources (infrastructure and personnel) within the network. We acknowledge, however, that our decision to use open source software and implement the platform from scratch was only feasible because our IT organization already runs its own data centers and has developer as well as development operations experience. Nevertheless, the global trend toward platform-as-a-service providers (hyperscalers) for storage and compute tasks poses challenges in recruiting experienced staff for on-premise installations. This presents a pain point for hospital networks: regulatory requirements require on-premise data storage, while most other sectors move to the cloud.

High levels of automation and agile development, characterized by short feature cycles and consistent data quality improvements, are necessary for user satisfaction. However, achieving swift project rollout and overall success necessitates continuous organizational investment and subject matter expert support.

Future developments will involve the extraction of knowledge from unstructured text, and we have collected preliminary findings on document pseudonymization [40]. Building research data platforms is not a purpose in itself. Meaningful research conducted on these platforms is needed to justify the collection of EHR data, even with patient consent. We are looking forward to research reports beyond the technical level on projects realized with our implementation.

## Supplementary material

10.2196/69853 Checklist 1 iCHECK-DH checklist.

## Abbreviations

API: application programming interface
CI/CD: continuous integration and continuous deployment
ED: emergency department
EHR: electronic health record
FHIR: Fast Healthcare Interoperability Resources
GDPR: general data privacy regulation
HDFS: Hadoop File System
HL7: Health Level Seven
ICU: intensive care unit
IHE: Integrating the Healthcare Enterprise
LOINC: Logical Observation Identifiers Names and Codes
MPI: master patient index
UAC: Usage and Access Committee
