# Supplementary material for: Lessons Learned From Building a Data Platform for Longitudinal, Analytical Use Cases and Scaling to 77 German Hospitals: Implementation Report
Source: JMIR Med Inform. 2025 Sep 12;13:e69853. doi: 10.2196/69853 (PMC12431789; doi:10.2196/69853)
Supplement: Checklist 1 [file medinform-v13-e69853-s001.docx]

Checklist of iCHECK-DH guidelines. iCHECK-DH: Guidelines and Checklist for the Reporting on Digital Health Implementations.

| section |  | Item | DESCRIPTION | Reference TO TEXT |
| --- | --- | --- | --- | --- |
| Title | 1 | Title  (M^[[1]](#footnote-1)^) | The manuscript title explicitly identifies the paper as an *implementation report*, states the core intervention (*data platform for longitudinal, analytical use cases*), and specifies the scope (*77 German hospitals*) | TITLE |
| Abstract | 2 | Abstract  (M) | Structured abstract summarises Background, Objective, Methods, Results, Conclusions, and keywords; it reports main consent rates (eg 1,475,244 approaches; 1,023,633 consents, 70.2 % consent rate), design (lambda architecture, decentralized connectors), and key lessons learned (agile, automation). | ABSTRACT |
| Introduction | 3 | Context  (M) | Setting: Helios Hospital Group, Germany; 77 acute-care hospitals plus outpatient centres. Contextual factors: heterogeneous EHR vendors, GDPR-driven on-premise requirement, alignment with national Medical Informatics Initiative (MI-I). | INTRODUCTION |
|  | 4 | Problem statement  (M) | Secondary use of EHR data outside university hospitals is limited; data silos impede cross-institutional analyses. Intervention addresses the lack of standardised, consent-based RWD from non-academic hospitals. | INTRODUCTION |
|  | 5 | Similar Interventions  (M) | Builds on MI-I concepts and prior single-site warehouses (e.g. HiGHmed, OMOP/i2b2) but is first large-scale private-network deployment with centralised on-premise Hadoop stack and unified broad-consent workflow. | INTRODUCTION + DISCUSSION |
| Methods | 6 | Aims and Objectives  (M) | implement a cross-institutional, multi-domain research data platform and describe lessons learned and unintended consequences. | METHODS: Aims and Objective |
|  | 7 | Blueprint summary  (M) | Four-layer lambda architecture (raw, transformation, consumption, data marts) on kerberised Hadoop; CONNECTOR appliances for minimisation/pseudonymisation; probabilistic MPI via IHE PIX; CI/CD-deployed Spark pipelines; governance via UAC + Atlas. | METHODS: Infrastructure and Software; Data-Modelling and Transformation; Governance |
|  | 8 | Technical Design  (M) | Technology stack: Hadoop distribution (HDFS, Hive/Impala, HBase), Spark, Airflow, Apache Hue; containerised Python/R; BI dashboards. CONNECTOR (Linux, Java/Python) at each site; high-availability PIX API fronting MPI. Rationale: on-premise compliance, open-source flexibility. | METHODS: Infrastructure and Software; ETL |
|  | 9 | Target  (M) | a) Organisational targets: hospitals able to embed consent workflow and provide interfaces.  (b) Data subjects: all admitted patients; inclusion requires written opt-in.  (c) End-users: researchers, quality managers, data scientists. | METHODS: Consen Management; AIMS AND OBJECTIVE |
|  | 10 | Data  (M) | Lifecycle: collection in source EHRs → minimisation/pseudonymisation in CONNECTOR → storage in secure, kerberised Hadoop cluster → project-specific second-layer pseudonymisation → researcher access governed by UAC. Ownership: hospitals; withdrawal triggers record suppression. Legal basis: GDPR + national secondary-use guidelines. | METHODS: Data minimization and pseudonymization, Governance |
|  | 11 | Interoperability  (M) | Standards: HL7 v2, FHIR bundles, IHE PIX, LOINC/UCUM, MI-I core dataset alignment. Raw-data-first policy preserves semantics; transformations iteratively harmonise to internal core model. | METHODS: ETL; Data modelling and transformation; LESSONS LEARNED:Interoperability & standardisation |
|  | 12 | Participating entities  (M) | Implementer/owner: Helios Hospital Group (private provider).  Internal partners: IT, research, compliance, project management teams.  External: no commercial vendor beyond Hadoop distribution; alignment (not funding) with German MI-I. Funding: internal operational budget. | METHODS: Requirements Engineering; Infrastructure and software |
|  | 13 | Budget Planning  (M) | Absolute figures undisclosed; costs included personnel (DevOps, analysts, trainers), additional cluster node, CONNECTOR appliances, consent UI integration, and maintenance. Timeline: 24-month roll-out → ongoing OPEX | LESSONS LEARNED: Infrastructure Scaling |
|  | 14 | Sustainability  (M) | Financed via routine IT budgets; platform embedded in core operations supporting QA and research deliverables. No reliance on temporary grants; on-premise infrastructure ensures long-term control. | DISCUSSION |
| RESULTS | 15 | Coverage  (M) | Geographic: national (Germany) private network.  Institutional: 77/89 hospitals integrated.  Patient coverage: 1,475,244 approaches in 24 months; 70.2 % consent; profile skew—70 % planned admissions, 21 % unplanned ED, 9 % outpatient ED. | RESULTS: Consent Management |
|  | 16 | Outcomes  (M) | Implementation outcomes: successful technical scale-up; consent uptake; data-volume and throughput metrics; automated data-quality framework; interactive data catalog; DVC-based reproducibility. No patient health-outcome evaluation in scope. | RESULTS: Data Volume; Data modelling and transformation |
|  | 17 | Lessons learned  (M) | Enablers: existing data-centre expertise, agile iterations, high automation, phased integration, raw-data-first, two-phase consent → data. Challenges: MPI load spikes, heterogenous EHR semantics, organisational effort for bedside consent, scaling HL7/IHE pipelines. Recommendations: start small, automate, decouple consent from admission where feasible, continuously monitor bias. | LESSONS LEARNED |
|  |  |  |  |  |
|  | 18 | Unintended consequences  (NM^[[2]](#footnote-2)^) | Selection bias toward planned admissions; under-representation of ED and vulnerable populations; potential algorithmic bias; technical side-effect: MPI time-outs during admission peaks. Mitigations: admission-type tagging, bias reporting requirements, exploring delayed consent. | UNINTENDED CONSEQUENCES |
| Discussion | 19 | Conclusion  (M) | Agile, consent-based data platform across 77 hospitals is feasible; high consent rates possible with organisational investment; interoperability and bias remain ongoing challenges; future work will address unstructured-text extraction. | DISCUSSION |
| General | 20 | General  (NM) | Ethics/governance: UAC review; ethics approval required for re-identification. Trial registration: not applicable. Funding: internal; conflicts of interest: none declared. |  |

1. M: Mandatory item [↑](#footnote-ref-1)
2. NM : Non-mandatory item [↑](#footnote-ref-2)
